# How to Define an Author? Awareness of Authorship Criteria

**DOI:** 10.5812/aapm.10877

**Published:** 2013-07-01

**Authors:** Mahsa Ghajarzadeh, Mehdi Mohammadifar, Saeid Safari

**Affiliations:** 1 Brain and Spinal Injury Repair Research Center (BASIR), Tehran University of Medical Sciences, Tehran, Iran; 2 Department of Anesthesiology, Rasoul Akram Medical Center, Iran University of Medical Sciences (IUMS), Tehran, Iran

**Keywords:** Authorship, Ethics, Publications

Dear Editor, 

Recognition as an author is an honor and credit for most researchers. In recent years, the number of authors has been increased along with the increase in the number of research articles (1). For instance, the mean number of authors per paper was 4.5 in 1980 and 6.9 in 2002 in biomedical journals (2). We know that, publication of a scientific work is the final stage of a long time careful planning, execution, analyses and scientific writing. So it is important to have articles without any scientific misconduct problems. Today, scientific misconduct is a crucial issue in the field of medical research. One of the most challenging issues in the field of scientific misconduct is disobeyed authorship criteria. According to ICMJE (International Committee of Medical Journal Editors) criteria, authorship credit should be based on the following three criteria:

1) Substantial contribution to the conception and design, data acquisition, or analysis and interpretation of data;

2) Drafting or revising the manuscript, critically for important intellectual content

3) Final approval of the version to be published (3).

A researcher should meet all criteria to be named as an author. Two terms should be considered according to this criteria, guest and ghost authors. Guest author is someone who is named as an author but without adequate contribution and ghost author is someone who had enough contribution while her/his name was not mentioned as an author (4, 5). Although many biomedical journals have suggested ICMJE criteria reading for all authors before article submission, it is evident that fewer authors are familiar with these criteria. Previous studies which evaluated fulfillment of authorship criteria in different journal articles revealed that existence of guest authors ranged from 64-74% (6-9). In a study evaluating fulfillment of authorship criteria among Iranian researchers, we found that only 62.8% of 296 authors met the criteria, 37% (110) authors identified as guest authors and two were considered to be ghost authors. So, it is important to make researchers familiar with authorship criteria and make them to obey ethical issues to reduce rate of dishonesty in medical reaches.
